# Mapping anatomical related entities to human body parts based on wikipedia in discharge summaries

**DOI:** 10.1186/s12859-019-3005-0

**Published:** 2019-08-17

**Authors:** Yipei Wang, Xingyu Fan, Luoxin Chen, Eric I-Chao Chang, Sophia Ananiadou, Junichi Tsujii, Yan Xu

**Affiliations:** 1 State Key Laboratory of Software Development Environment and Key Laboratory of Biomechanics and Mechanobiology of Ministry of Education and Research Institute of Beihang University in Shenzhen, Beijing Advanced Innovation Center for Biomedical Engineering, Beihang University, Xueyuan Road No.37, Beijing, 100191 China; 2 Bioengineering College of Chongqing University, Shazheng Street No. 174, Chongqing, 400044 China; 3 Microsoft Research, Danling Street No. 5, Beijing, 100080 China; 4 The National Centre for Text Mining, School of Computer Science, The University of Manchester, Manchester, UK; 5 Artificial Intelligence Research Center (AIRC), Tokyo, Japan

**Keywords:** Natural language processing, Anatomical entity, Human body parts, Named entity normalization, Wikipedia

## Abstract

***:**

Background Consisting of dictated free-text documents such as discharge summaries, medical narratives are widely used in medical natural language processing. Relationships between anatomical entities and human body parts are crucial for building medical text mining applications. To achieve this, we establish a mapping system consisting of a Wikipedia-based scoring algorithm and a named entity normalization method (NEN). The mapping system makes full use of information available on Wikipedia, which is a comprehensive Internet medical knowledge base. We also built a new ontology, Tree of Human Body Parts (THBP), from core anatomical parts by referring to anatomical experts and Unified Medical Language Systems (UMLS) to make the mapping system efficacious for clinical treatments.

***:**

Result The gold standard is derived from 50 discharge summaries from our previous work, in which 2,224 anatomical entities are included. The F1-measure of the baseline system is 70.20%, while our algorithm based on Wikipedia achieves 86.67% with the assistance of NEN.

***:**

Conclusions We construct a framework to map anatomical entities to THBP ontology using normalization and a scoring algorithm based on Wikipedia. The proposed framework is proven to be much more effective and efficient than the main baseline system.

## Background

A medical narrative which consists of dictated free-text documents such as discharge summaries is integral to clinical patient records. From databases of free-text medical narratives, a mapping system can be built to find relevant information about the human body parts for clinical and research purposes. Generally, human body parts can be classified according to physiology or body structure [1]. The classification result based on physiology shows its advantage in matching organs and tissues with similar functions, such as muscle classification in different body parts [2]. However, in some practical situations, especially in clinical cases, doctors first pay attention to the anatomical position rather than the functional relation of human body parts. For example, if a patient has chest pain, organs close to the position of chest such as the heart, lung, and esophagus [3], are all regarded as suspected causes despite that they belong to different physiological systems. Therefore, classification result according to body structure helps to locate possible causes. Moreover, the classification result based on position is compatible with the arrangement of hospital departments. For example, the otolaryngological department in a hospital is in charge of patients who have problems with their noses, ears, and throats. Therefore, we classify human body parts according to body structure and construct the ontology from a dictionary of anatomical locations to build the mapping system, which shows clear subordinate relations of human body parts.

One challenge of designing the mapping system is the diverse forms of an anatomical entities. Named entities related to body parts, organs, and their subparts are defined as anatomical entities [4]. In this paper, we use the phrase “anatomical related entities” to denote named entities and their related medical terms, including problems, medical tests, and treatments. Named entities that directly denote human body parts are considered explicit anatomical related entities (e.g., colon). Name entities that do not refer to but are highly related to human body parts are defined as implicit anatomical related entities (e.g., colonoscopy). In our previous research [5], a Conditional Random Fields (CRF) model was presented for anatomical entity recognition. Entities from discharge summaries are recognized, extracted, and labeled as explicit or implicit entities. One limitation to this paper is that anatomical entities are only extracted and classified into two clusters (explicit anatomical entities and implicit anatomical entities). It cannot be applied in practice since meanings of the extracted anatomical entities cannot be easily discerned in the presence of various forms or abbreviations of entities. The extracted entities are of great value in clinical text mining if they are properly organized following the structure of related body parts. Therefore, attention has been focused on detecting and utilizing relationships between entities extracted in the previous paper and anatomical parts to map anatomical entities to the specific human body parts to which they belong.

For explicit anatomical related entities, there are dictionary-based methods (e.g., string-matching) that can conduct mapping. However, quite a few implicit anatomical related entities cannot be mapped to certain body parts by string-matching methods. The reason is that there is no common substring between the entities and the body parts. For example, the following two sentences come from a discharge summary:*“Given this, it was advised that the patient have a colonoscopy to rule out further bleeding”.“The patient underwent a flex sigmoidoscopy on Friday, 11-02”.*If we use string-matching, colonoscopy which is a medical test can be mapped to colon because of the common string “colon”. However, “sigmoidoscopy”, which is also a medical test related to colon, cannot be mapped to “colon” since the string “sigmoidoscopy” does not contain the substring “colon”. In Systematized Nomenclature of Medicine - Clinical Term (SNOMED-CT) [6], several entities are mapped to more than one terms. Therefore, although there exists some implicit relationships in SNOMED-CT, it is difficult to distinguish the exact concept for one entity in one certain context from more than one matched terms. The highest results for normalizing entities from clinical documents to SNOMED-CT in a recent competition was 0.757 [7], indicating the complexity of this task. Without correct matching to ontology, such relationships are difficult to extract and utilize.

In addition to implicit anatomical related entities, abbreviations, which are common in medical records, are also difficult to retrieve using string-matching. Since the abbreviations of anatomical related entities in medical records are not always the same, it is not easy to build a supporting dictionary to cover all medical abbreviations. For example, “extremity” has abbreviations such as “ext”, “extrem”, “ue” (upper extremity), and “le” (lower extremity). There are too many synonyms in medical texts which are difficult to be recognized using limited dictionaries.

The key to solving these issues is to identify the standard form or the related anatomical part for each entity. A named entity normalization (NEN) method is presented in this work to explore the relationships between anatomical related entities and human body parts. The relationships recognized by NEN can locate the original or correlated body parts of implicit anatomical related entities and abbreviations, which makes mapping easier. In the NEN system, extensive information results in a high level of effectiveness [8]. Thus, an external knowledge base is required to expand sources of information and to avoid the limitations of dictionaries. Wikipedia, one of the most comprehensive databases for medical informatics [9, 10], is selected for this work. Each anatomical related entity is first searched in Wikipedia to obtain its explanation. After that, all human body parts appeared in the explanation of the entity are chosen as candidates. Finally, a special scoring algorithm is applied to determine which candidates are the final normalization results that later be mapped to an ontology.

An ontology is an information aggregation made up of standard forms, properties, and relationships of entities [11]. The ontology is key to eliciting available information from a knowledge base. Naturally, the quality of the ontology is also a factor that greatly influences the performance of the mapping system. A common problem is identifying the proper concept for one entity because a large amount of string-overlap exists in these hierarchies. Established standard vocabularies (e.g., UMLS, MeSH) are too complicated for mapping or clinical work because they contain excessively detailed human body parts. In UMLS, more than 60,000 human body locations are placed in a group called “Body Location or Region” and no specific subsets are constructed; UMLS is thus not very efficient in clinical work because it is not well-classified. In addition, some string overlaps among concepts may cause confusions. In SNOMED-CT, several concepts might be detected for one entity by string matching method. We randomly picked several concepts and looked them up in UMLS for relevant concepts. For example, there are more than 3,000 concepts in SNOMED-CT contain the term “pain”. It is therefore difficult to determine which entity is the one that is needed. Even when special attention is paid to this task, the effect seems to be insignificant. In Task 1 of SemEval-2015, 16 teams created complicated systems to recognize proper concepts, but even the best-performing team could achieve only a 0.757 F1-measure [7], which does not meet the standard of clinical use and cannot be applied to clinical practice. To solve such problems, a new ontology simplified from existing medical databases (e.g., UMLS) for discharge summaries is required.

In this study, we developed a new ontology called THBP using standardized names of human body parts extracted from UMLS with the help of three clinical experts, provided a set of anatomy annotations from 50 discharge summaries, and successfully built a mapping system to map anatomical entities in discharge summaries automatically. In this new ontology, human body parts are organized by their location. Therefore, THBP is more convenient for clinical text mining applications. The main contributions of this study are threefold. 1) We are the first to successfully map anatomical related entities from discharge summaries directly to human body parts. 2) We develop a highly accurate approach to this problem by combining normalization and Wiki-based algorithms. 3) Finally, we organize THBP as an ontology for mapping anatomical named entities, which will be published open source on the Internet.

## Related work

Anatomy is one of the foundations of modern medicine and greatly contributes to medical research as well as clinical diagnoses. Therefore, building a semantic network of anatomical related entities is of great value for both experts and novices [12]. Much effort has been made on this research topic. Rosse and Mejino constructed a large semantic network of more than 100,000 terms that refer to anatomical entities called the Foundational Model of Anatomy (FMA) [13]. The interface of FMA, called Emily, has proven useful for most queries on anatomical relationships [14]. Niggemann et al. established a functional anatomical model for nervous systems [15]. Zhang et al. [16, 17] focused on improving the two-tree structure of The Unified Medical Language System’s (UMLS’s) Semantic Network (SN). These studies focused only on improving the model from a theoretical perspective without considering their clinical implications.

### Anatomical ontology

In clinical practices, medical tests and diagnoses are usually related to the locations of body parts with suffering, an anatomical network based on body positions is more effective and helpful. SNOMED-CT is an ontology that covers a large number of clinical concepts and relations, including anatomical parts, and demonstrates a high efficiency in dealing with several clinical research works [6, 18]. However, difficulties in concept identification, such as abbreviations and different forms of the same entity, undermine its functionality. No perfect solution for this has been determined even by experts in the field [7]. Another anatomical ontology, Uberon, contains anatomical concepts and links to other resources, such as Wikipedia [19, 20]. Unfortunately, this ontology is not specific for human anatomy. The intention was to build a bridge between anatomical structures for different species. Anatomical related entities that do not simply represent anatomical structures, such as sigmoidoscopy, do not have labels and do not exist in the expanded content of related anatomical parts, making them unavailable for some issues in human anatomy. Each ontology has its own limits and cannot serve all literature mining tasks. Consequently, a compendious ontology extracted from existing ones is recommended for solving these special tasks.

### Named entity normalization

Named entity normalization builds a mapping relationship between named entities in text and ontology. Since standardized vocabularies and terminologies are essential in medical information processing and normalization [21], several predefined vocabularies have been built, such as Gene Ontology [22], Entrez Gene [23], Medical Subject Headings (MeSH) [24], and the Unified Medical Language System (UMLS) Metathesaurus [25]. In the biomedical domain, earlier works have tried to build a normalization system based on these vocabularies. In 1988, Elkins et al. mapped named entities in narrative texts to MeSH [26]. Aronson [27, 28] developed a program, called MetaMap, to map biomedical text to the UMLS Metathesaurus in 2001. Also in 2001, Mutalik et al. [29] developed a program, Negfinder, to detect negated concepts of UMLS Metathesaurus in medical narratives. Xuan et al. [30] developed PubAnatomy to discern relationships between molecular level data and neuroanatomical structures from cross-domain data. In 2007, Schuemie et al. [31] developed a gene name normalization system called Peregrine with a simple dictionary lookup method and several following steps.

The works discussed above apply dictionary-based methods and have achieved considerable progress. Nevertheless, such early investigations simply apply lexical matching, and the accuracy of these systems depends heavily on the quality of both technology and the text. As a result, for dictionary-based approaches, without being assigned with good text and a good dictionary, these methods would not achieve high accuracy, especially recall. Particularly in clinical texts, abbreviations are very common and difficult to be fully included in one dictionary.

To address the above-mentioned problems, research interests have recently moved to using NLP and statistical machine learning methods to solve normalization problems [32, 33]. Kang et al. compared MetaMap with Peregrine on the Arizona Disease Corpus, and attached an NLP module to them, which helped to improve normalization accuracy [34]. Kovačević et al. focused on extraction and normalization of temporal expressions using rules and machine learning approaches such as Conditional Random Fields (CRF) [35]. Wang et al. utilized MedLEE and designed a statistical model to detect medical events and entities potentially related to adverse drug events for pharmacovigilance purposes [36]. Penalized logistic regression was applied in Li’s study to determine adverse drug reactions [37]. Leaman et al. conducted a series of research regarding named entity recognition and normalization. They first presented a method that exploited pairwise learning to uncover diseases mentioned in a snippet of text, which is termed disease name normalization (DNorm) [38]. After that, the tmChem system [39] was proposed to locate chemical named entities in texts. The system combined two CRF models, a modification of BANNER [40] and a CRF model based on the tmVar system [41], in one ensemble. Robert Leaman and Zhiyong Lu then introduced a joint named entity recognition (NER) and normalization model which includes a semi-Markov classifier and a rich feature approach to conducting NER and semantic indexing [42]. A Java implementation of this model called TaggerOne was also presented as a universal toolkit for all types of entities. Soysal et al. developed a clinical NLP toolkit called CLAMP (Clinical Language Annotation, Modeling, and Processing) [43] that offers users a user-friendly graphical interface with high-performance NLP modules to establish customized NLP pipelines that solve their own problems.

Overall, statistical methods have achieved great success in normalizing entities and detecting relationships, particularly for clinical texts. Since our work is built on clinical texts (i.e., discharge summaries) and we need to identify a huge number of abbreviations, we applied machine learning methods to normalization for anatomical related entities.

### Wikipedia as an external knowledge base

When building an effective normalization system, employing a large external knowledge base has a positive impact on the performance of the whole system. Wikipedia is selected as the external knowledge base in this study, considering that Wikipedia is regarded as a valuable knowledge base in medical language processing [9]. Information on Wikipedia is more abundant than established biomedical dictionaries without limitations on quantity and is updated frequently. Its information also has a higher level of accuracy than most other references [10]. These features make Wikipedia a valuable knowledge base for medical normalization. For example, Rink et al. developed a relation extraction system of clinical terms by utilizing Wikipedia in 2011 [44], while several other biomedical NLP research efforts have benefited from Wikipedia [45–49].

## Methods

### Materials

#### The creation of tree of human body parts

Facing the difficulty of directly exploiting existing ontologies, we decided to organize a compendious but specialized ontology that is consistent with the structure of established ontologies and compatible with the arrangement of hospital departments. To accomplish this task, entities from the “Body Location or Region” group in UMLS are first extracted. The essential clinical body parts are then chosen according to clinical experts and later be used for the building the THBP. Finally, we get inspiration from FMA and SNOMED-CT and discuss the structure of the new ontology with doctors. These all ensure that the framework of the new ontology is aligned with those ontologies and the arrangement of the departments in a hospital.

**Fig. 1:**
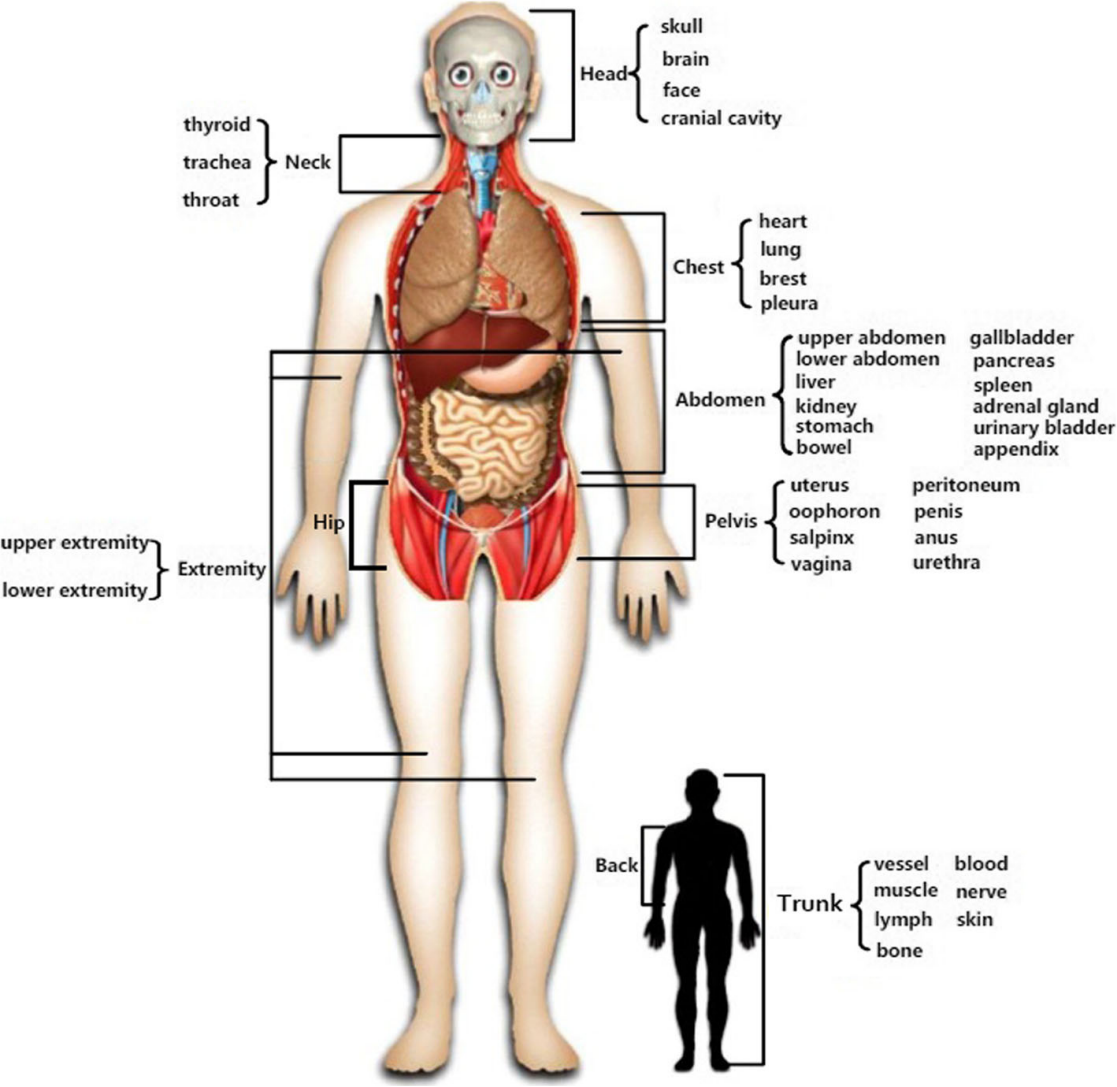
The top level of the Tree of Human Body Parts (THBP) THBP consists of 9 parts: head, neck, chest, abdomen, pelvis, back, hip, extremity, and trunk. For each part, its sublayer is constructed by organs or tissues in this part. The human body image in the figure was created by the author

Anatomical structures of humans can be classified into five levels - systems, organs, tissue, cells, and chemicals (e.g., molecules or ions) [1]. Since cells and chemicals are countless and exist in every part of the human body, it is unnecessary to map them to particular human body parts in this case. Therefore, we only focus on the former three levels of anatomical structures. According to anatomy studies [50], human body parts can be divided into nine different parts by position: the head, neck, chest, abdomen, pelvis, back, hip, extremity, and trunk. In our ontology, the nine anatomical positions are regarded as the top level. Each part contains anatomical structures that belong to that position as subordinates in lower levels. This hierarchical classification structure is defined as THBP. Details are shown in Fig. 1.

Anatomical related entities usually have several synonyms and abbreviations in biomedical text. For example, “abdomen”, “peritoneal cavity”, and “abd” all refer to the same body part. For body parts that are called by more than one terms, one alias is treated as the formal name and other aliases including abbreviations and synonyms in the THBP are considered supplemental. For example, “abdomen” is chosen as the formal name for that body part and the others are supplemental (e.g., abdomen – abd – abdn – peritoneal cavity – abdominal cavity – enterocoelia).

#### Annotation

Our annotation dataset comes from the output of our previous work [5], which used a CRF model to recognize anatomical named entities from 300 discharge summaries in the 2010 i2b2 challenge corpus [51]. The discharge summaries are provided by Partners Healthcare, Beth Israel Deaconess Medical Center, and the University of Pittsburgh Medical Center. Since annotation quality has a significant impact on the whole system, several reiterations of preliminary annotation are conducted to build the annotation guideline before the final annotation. There are three annotators, two of whom have biomedical engineering background with human anatomy expertise while the third has a clinical background. 10 discharge summaries are randomly selected from our previous work for preliminary annotation. 50 discharge summaries randomly selected from the previous work and 2,224 anatomical related entities with their annotations are extracted as the final annotation dataset of this study.

#### Annotation guideline

An easily followed standard guideline is required to ensure consistency and help annotators work independently. The process of building the guideline follows the Delphi method [52]. Guidelines are built and unified from a development set of articles.

To build the guideline, the annotators first make annotations on 10 discharge summaries separately and then find the differences between these annotations through discussion. Note that these 10 discharge summaries from our previous work are only used for building the annotation guideline and are not included in the experiment annotation dataset. Based on their discussion and further studies of references, each annotator constructs their own list of guidelines. Revisions on annotation are made independently following their own guidelines. Then there is another discussion. The cycle of holding discussions, amending guidelines, and revising annotations is repeated until a convincing agreement is reached. Finally, three sets of mutually independent guidelines are compiled into a unified one.

The annotation results of anatomical named entities follow the rules below. Note that “positional words” in the following context refers to prefixes or words that indicating the location of human body parts. We defined some necessary components based on anthropotomy knowledge and then match them with the most relevant concept in UMLS.

1. All anatomical named entities are normalized by position instead of function (e.g., “carotid artery” is normalized to “neck”, not “artery”). This is in accordance with the idea of creating an ontology that based on position. The trunk is considered the “stem” of the human body, including organs and tissues that are distributed across the whole body, such as nerves, blood, and bones.

2. The mapping results of anatomical named entities are the lowest layers of THBP. For example, the mitral valve is located in the chest and is part of the heart. Considering that the “heart” is a sublayer of the “chest”, the mitral valve is mapped to the heart instead of the chest.

3. For anatomical related entities that are exact parts of THBP (e.g., heart), the mapping results are their original forms. If the full name of an abbreviation can be found in THBP, the abbreviation is normalized to the full name (e.g., “EXT” to “extremity”).

4. Abbreviations consisting of several different anatomical locations are mapped to several different parts of THBP. For example, HEENT as an abbreviation of Head–Eye–Ear–Nose–Throat is mapped to “head”, “eye”, “ear”, “nose”, and “throat”.

5. The mapping results are in singular form, while the positional prefixes or words in named entities remain the same. For instance, “left extremities” is mapped to “left extremity”.

#### Annotation flow

The articles in the experiment set are annotated based on the unified guidelines mentioned above. Two rounds of annotations are carried out on all 50 discharge summaries. In the first round, two annotators (A1 and A2) with biomedical engineering backgrounds annotate the same discharge summary independently. When there are disagreements between their results, the third annotator with a clinical background (A3) acts as referee and makes the final decision. After that, A3 explains the reasons for the judgments and the three annotators discuss the guidelines. During this discussion, guidelines are revised for one last time. In the second round, A1 and A2 annotate 50 discharge summaries according to the final version of guidelines. When A1 and A2 disagrees, A3 makes the final decision. For example, “bronchitis” is an entity that belongs to “neck” and is related to “trachea”, a sub-layer of “neck”. If there is a disagreement between A1 and A2 in this situation, A3 will point out that it should be normalized to the lowest related layer “trachea” according to Rule 2 above. The final result, therefore, is “trachea”.

#### Inter-annotator agreement

To determine whether the annotation results can be used as the gold standard, the inter-annotator agreement is measured by F1-scores. Table 1 shows comparisons between the annotation results of A1 and A2. Suppose the result of A1 is the ground truth, we calculate the precision, recall and F1 score of A2. Table 2 displays the inter-annotator agreement, which compares the results of each annotation with the final annotation results.

**Table 1:** Inter-annotator agreement between A1 and A2

Annotator	Precision	Recall	F1
A1 and A2	89.93%	91.34%	90.63%

**Table 2:** Inter-annotator agreement between each annotator and the gold standard

Annotator	Precision	Recall	F1
A1	89.53%	88.63%	89.07%
A2	90.28%	91.88%	91.07%

According to Table 1 and Table 2, in the second round, there are still minor disagreements between the annotations of A1 and A2 or between their annotations with the gold standard. There are some possible reasons for the small disagreement. First, some anatomical named entities (especially diseases) are related to several different anatomical locations, which makes it difficult to determine which branch of THBP they belong to. In addition, several abbreviations have multiple complete forms, causing disagreements on determining which label should be assigned to this abbreviation. Furthermore, each annotator is partial to her/his own guideline and ignores some anatomical named entities while the other annotator might annotate the ones s/he ignores. However, since all the differences are small and the mistakes made by A1 and A2 could be corrected by A3, the results can be treated as the gold standard for the following experiments.

### Algorithms

**Fig. 2:**
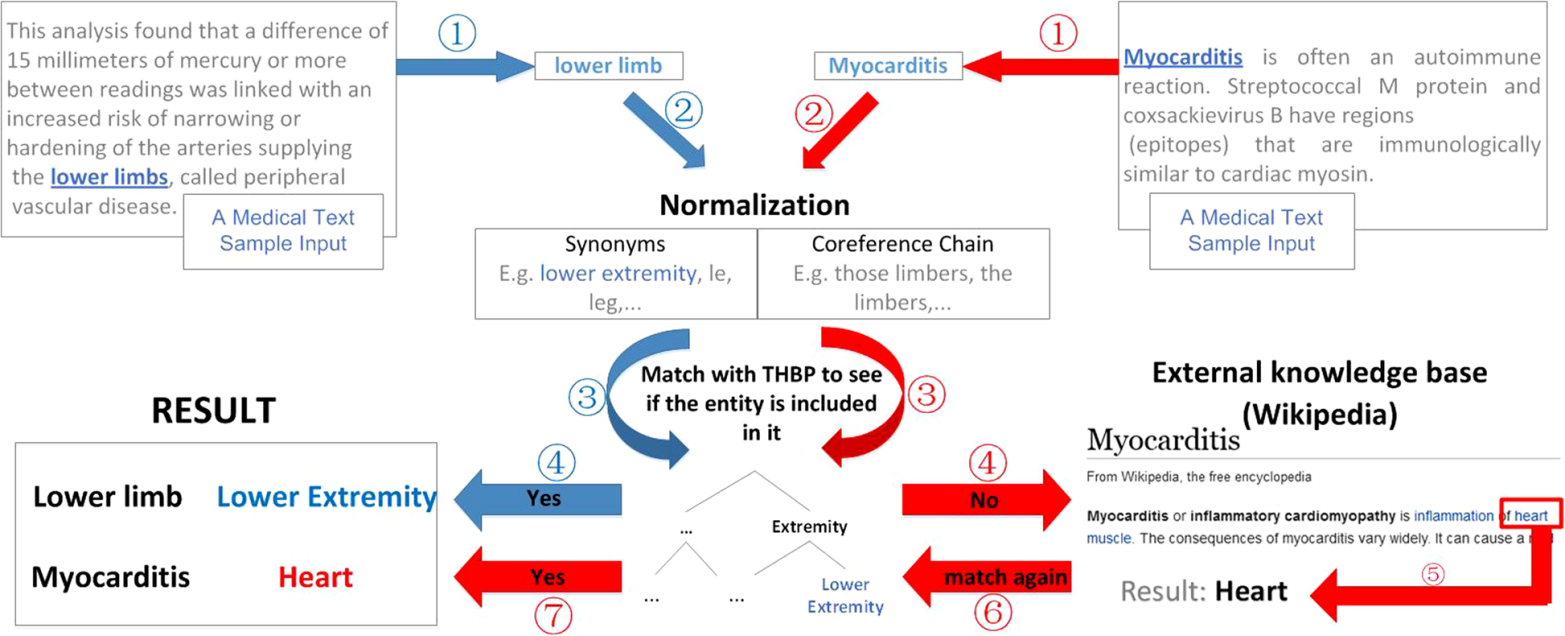
The flowchart of mapping. Anatomical related entities are extracted from medical text first. Then the entities are normalized by using our synonyms dictionary or co-reference chains provided in the corpus of [51]. After that, we match the normalized entities with THBP to see if they are included. If the entities are successfully matched (e.g. lower limb), the results are considered final. If not (e.g. myocarditis), it turns to the external knowledge base to normalize entities and match them with THBP again

To accomplish the mapping task, we present the result of combining these methods: the string-matching method (baseline system), named entity normalization, and the exploitation of Wikipedia (including Wikipedia distance scoring and frequency scoring). The flowchart is shown in Fig. 2.

### Baseline system

**Fig. 3:**
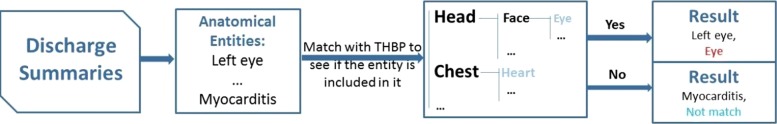
The baseline system. First, anatomical related entities are extracted from medical records. After that, entities are matched with THBP to see if they are included. If so, an entity such as left eye, which is included in eye, the result is that left eye belongs to the class of eye. Otherwise (e.g. Myocarditis), the result comes out as not matched

We use string matching algorithm as our baseline system. Entities are extracted from discharge summaries and directly mapped to our ontology THBP through a string-matching method (shown in Fig. 3). A porter stemmer [53] is used for stemming. However, the same entity in different forms cannot be recognized in this system, e.g., “heart disease” and “coronary artery disease”.

### Named entity normalization

Anatomical named entities in biomedical text and Wikipedia explanations have a diverse array of forms, while entities in the ontology and Wikipedia entries are all standardized. As a result, only entities in normalized form can be correctly mapped. To map the named entities to the ontology and retrieve Wikipedia entries, anatomical named entities need to be normalized. Words in plural form are changed to singular form, and stop words in anatomical named entities are removed.

Abbreviations are common in biomedical text and affect mapping performance. But the method mentioned above is not applicable to abbreviation normalization. To normalize abbreviations, two approaches are explored. Inspired by the success of previous works [54, 55], a synonym dictionary extracted from these works and Wikipedia is built to map common abbreviations. In addition, several studies have shown that full forms of some abbreviations, called local abbreviations, exist in the same medical record, and co-reference relations between local abbreviations and their full forms can be easily detected [51, 56]. Therefore, these co-reference relations can be discovered and applied to normalize local abbreviations. In this work, co-reference chains, which have already been annotated in the i2b2 corpus, are used to obtain the original full forms of abbreviations [51].

Positional words provide informative knowledge about patients for clinical practitioners. Hence, they are preserved while annotating anatomical named entities and building the tree of human body parts. A dictionary is built to distinguish positional words. Positional words in the Biological Spatial Ontology [57] are also added. The positional words are removed before normalization and added to the normalization results in an appropriate way. For example, “legs bilaterally” is processed into “legs” to normalize it, and the normalization result is “lower extremity”. Consequently, the positional word “bilaterally” is added to “lower extremity”, and the final result of “legs bilaterally” is “bilateral lower extremity”.

### Wikipedia scoring algorithm

The aim of our task is to extract the exact common semantic parts which belong to the same anatomical location among different classes of entities in the ontology. An NEN system is suitable for this task, but its accuracy is greatly limited by the knowledge base. In an NEN task, a reliable external knowledge base would enhance the performance of the normalization system [8]. Wikipedia, considered a key tool in the medical field [9, 10], has been proven comprehensive and accurate in prior studies [44–49, 58]. Therefore, we chose Wikipedia as the external knowledge base in this system. We design the scoring algorithms to exploit the extra information for each named entity provided by Wikipedia. We first use API to search terms and apply stemming to all words in the discharge summaries before matching.

Entries in Wikipedia provide users with detailed information about anatomical named entities, such as the explanations of diseases or functional regions of treatments, which are necessary for normalization. In the Wikipedia explanation of each anatomical named entity, there are several related anatomical locations in the context. It is reasonable to assume that the normalization result of the anatomical named entity is the counterpart of an anatomical location in THBP, which appears in the explanation context. For instance, in the Wikipedia explanation of “electrocardiography”, there are anatomical locations such as “heart”, “chest”, “thorax”, etc. As “electrocardiography” is a test to examine the function of the heart, the result of this implicit anatomical named entity should be “heart”.

Inspired by previous works [59–61], frequency (i.e., the number of times one anatomical location appears in the context) and distance (i.e., the average distance between each appearance of the anatomical location and the onset of the explanation) are considered as the two main factors in determining the interdependency of anatomical locations in context and the anatomical named entity. Generally, the higher the frequency of an anatomical location, the more related it is to the named entity [59, 60]; the smaller the distance of an anatomical location is, the more related it is to the named entity [61]. Therefore, we designed the scoring algorithms based on distance and frequency.

**Algorithm 1: Based on distance** Considering that words close to each other in text are related in semantics [61], the word distance in Wikipedia explanations can represent the correlation between entries and entities in texts. Therefore, we assume that the earlier anatomical related entities appear in an explanation, the closer they are to the entry. To normalize the distances to be equally compared, the following formulas are listed to score the distance:

1 $$ Score =\left\{ \begin{array}{lll} \cos (D(n)\times & \frac{\pi }{2}/\max(D)) & \max(D)> 1 \\ &1 & \max(D)=1 \\ &0 & \max(D)=0 \end{array}\right.  $$

*D*(*n*) denotes the number of strings from the beginning of entry to the anatomical named entity which can be matched by THBP. max(*D*) represents the distance of last matched anatomical named entity.

In this way, the distance is transformed to a cosine similarity ranging from 0 to 1.

**Algorithm 2: Based on frequency** In addition to distance, as stated in [59, 60], the number of times of an anatomical related entity appears in the explanation of an entry also represents the correlation between them. The score of each matched anatomical named entity is calculated by the following formulas:

2 $$ Score = \left\{\begin{array}{ll} 2.5 \times F(n) & first entity \\ \quad F(n) & others \end{array}\right.  $$

*F*(*n*) is the frequency of the anatomical named entity which can be matched by THBP. Considering the first appearing anatomical named entity that can be matched by THBP is strongly related to the mapping results, 2.5 is the multiplier to the frequency of the first appearing anatomical related entity. The multiplier 2.5 is determined through cross validation.

To verify the validity of our algorithms, 50 anatomical named entities and their Wikipedia entries are randomly extracted as test data, on which two Wiki-based algorithms are employed to obtain mapping results. Table 3 shows the mapping results.

**Table 3:** Inter-annotator agreement between A1 and A2

	Correct	Wrong	Accuracy
Distance	35	15	70.00%
Frequency	34	16	68.00%

Both algorithms achieve a fairly high level of accuracy, which proves the accuracy of our earlier assumption and the effectiveness of the algorithms. According to the results, both scoring strategies are beneficial to the task.

**Algorithm 3: Based on distance & frequency** After collecting the distance and frequency of each word, we combine the distance score and the frequency score to a final score. Scoring formulas are combined to grade each word so that the correlation is better represented. The formula is as follows:

3 $$ Score(n) = a \times f(D(n)) + b \times f(F(n))  $$

where

4 $$ f(D(n))=\cos (D(n)) \times \frac{\pi}{2} \times \max (D))  $$

5 $$ f(F(n))=F(n)  $$

To determine the importance of the first matched word in the entry, we multiply 2.5 by the frequency of the first named entity showing up in the entry. The constant 2.5 is determined by cross validation.

6 $$ f(F(n))=2.5 \times F(n)  $$

In the formula, *a* and *b* are coefficients of *f*(*D*(*n*)) and *f*(*F*(*n*)) respectively. *a*=15 and *b*=1 are selected which contribute to the best results based on cross validation.

After calculating the scores, we choose the word with the largest score to be the normalization result of the corresponding anatomical named entity.

## Results

### Experiment data

50 discharge summaries from the i2b2 Challenge [51] are used in our experiments, from which 2224 anatomical named entities are extracted [5]. F1-measure is used to evaluate the quality of each method on two series of experiments.

To compare the improvement of each component over the baseline framework, anatomical named entities are mapped by separately adding each method to the baseline framework. Since each component is independent in separate parts of the framework, a group of experiments is carried out to evaluate how the accumulation of methods can influence the final mapping result. Table 4 shows the results of separate components-based experiments and increments compared with baseline mapping results as well as the F1-scores and improvements of each step for accumulation experiments.

**Table 4:** Results of combinations of different methods with baseline

	Precision	Recall	F1	*Δ* F1
B	83.87%	60.37%	70.20%	-
B+N	86.87%	67.00%	75.65%	5.45%
B+D	86.16%	67.12%	75.46%	5.26%
B+F	86.24%	68.66%	76.45%	6.25%
B+D&F	86.51%	69.19%	76.89%	6.69%
B+N+D	88.48%	79.15%	83.55%	13.35%
B+N+F	88.47%	79.09%	83.52%	13.32%
B+N+D&F	89.11%	84.36%	86.67%	16.47%

Note: B-Baseline, N-Normalization, D-Distance, F-Frequency, D&F-Distance & Frequency

As Table 4 shows, Wikipedia individually achieves 76.89% with the final algorithm, which is higher than the baseline (70.20%) and normalization (75.65%). The result indicates that extracting anatomical information from Wikipedia contributes the most to the mapping performance.

In accumulation experiments, the final result of the whole mapping system stops increasing at 86.67%, while the distance-based algorithm and the frequency-based algorithm individually rise to 83.55% and 83.52% with the assistance of normalization. This result shows the primary contributions of the normalization system. With normalized entities in entries and explanations, information in Wikipedia can be better used.

The increase in the final algorithm (Baseline + Normalization + Distance & Frequency) compared to the Baseline is because the problem of synonyms, especially abbreviations, is solved. In Baseline, almost no abbreviations were matched. Even matching the entities directly to SNOMED-CT, 19.82% entities cannot be separately matched and 71.34% of them are abbreviations. In the results of the final algorithm, 53.12% of abbreviations that are not matched to SNOMED-CT are properly matched. These results show the significance of our algorithm especially in solving abbreviation problems.

## Dicussion

### Baseline system

SNOMED-CT was also one of the candidate ontologies for the baseline system. However, since SNOMED-CT is a post-coordinated terminology, it lacks some necessary relationships that are included in our ontology. Experiments also show that the performance of SNOMED-CT is inferior to string matching. Therefore, SNOMED-CT was discarded.

### Error analysis

Though significant progress is achieved by our mapping system, performance can be further improved. Four possible causes are provided below.

The first cause is the abbreviation method. By using several methods, the problem in normalizing abbreviations is alleviated to some extent. However, it is almost impossible to build a comprehensive abbreviation table because abbreviations are countless. Therefore, performance is still limited by abbreviations. Moreover, the same abbreviation may refer to multiple entities, which calls for a disambiguation system to solve the problem.

Second, there are special characters in some medical terms which are difficult to process. Both removing them and treating them as space can cause problems. For example, if simply removing special characters, the system will recognize “cad/chf” (coronary artery disease/congestive heart failure) as one entity. In another condition, treating them as space will normalize “hf-cells” (heart failure cells) to “cell”, which is not included in THBP, instead of “heart”. The corresponding anatomical locations cannot be found in either situation.

In addition, some anatomical named entities can be mapped to more than one anatomical locations, which causes another error in the mapping system. For example, “renal vein” can be mapped to “vein” and “kidney”. It makes sense in both locations. In our system, it is mapped only to “vein” instead of “kidney”.

Finally, some confusing anatomical named entities may appear in discharge summaries. For example, “right lower lobe” can refer to organs such as lung or liver. In annotation, annotators can determine which anatomical location the named entity is mapped to by looking at context. However, the mapping system cannot predict the anatomical location by linking context, which is one of the sources of error.

### Future work

Considering that the abbreviation is a major source of influence to test errors, a more sophisticated abbreviation normalization system is expected to bring significant improvement. As stated above, the same abbreviation might refer to different anatomical related entities, thus introducing errors. A disambiguation system would assist an NEN system to reduce such errors and to find the correct meaning of abbreviations. We could improve our performance in the future research by utilizing a word disambiguation system. In addition, we will also consider using definitions in UMLS Metathesaurus to further improve the performance.

## Conclusion

The THBP ontology can be used to successfully map and bridge different semantic classes of anatomical named entities and anatomical locations. To complete the mapping objective, a scoring algorithm is presented to make use of information on Wikipedia, while an NEN system is also added to improve performance. The results demonstrate that our methods successfully accomplish this task.

## Data Availability

The datasets generated during and/or analyzed during the current study, the ontology THBP and code are available at GitHub: https://github.com/xuyanbuaa/THBP.

## Abbreviations

CLAMP: Clinical Language Annotation, Modeling, and Processing
CRF: Conditional random field
FMA: Foundational Model of Anatomy
MeSH: Medical Subject Headings
NEN: named entity normalization
NER: named entity recognition
SNOMED-CT: Systematized Nomenclature of Medicine - Clinical Term
THBP: tree of human body parts
UMLS: Unified Medical Language Systems
